# A High Dietary Glycemic Index Increases Total Mortality in a Mediterranean Population at High Cardiovascular Risk

**DOI:** 10.1371/journal.pone.0107968

**Published:** 2014-09-24

**Authors:** Itandehui Castro-Quezada, Almudena Sánchez-Villegas, Ramón Estruch, Jordi Salas-Salvadó, Dolores Corella, Helmut Schröder, Jacqueline Álvarez-Pérez, María Dolores Ruiz-López, Reyes Artacho, Emilio Ros, Mónica Bulló, María-Isabel Covas, Valentina Ruiz-Gutiérrez, Miguel Ruiz-Canela, Pilar Buil-Cosiales, Enrique Gómez-Gracia, José Lapetra, Xavier Pintó, Fernando Arós, Miquel Fiol, Rosa María Lamuela-Raventós, Miguel Ángel Martínez-González, Lluís Serra-Majem, on behalf of the PREDIMED Study Investigators

**Affiliations:** 1 Research Institute of Biomedical and Health Sciences, University of Las Palmas de Gran Canaria, Las Palmas de Gran Canaria, Spain; 2 Ciber Fisiopatología Obesidad y Nutrición (CIBEROBN, CB06/03), Instituto de Salud Carlos III (ISCIII), Spanish Government, Madrid, Spain; 3 Department of Internal Medicine, Hospital Clinic, Institut d'Investigacions Biomèdiques August Pi Sunyer (IDIBAPS), Barcelona, Spain; 4 Human Nutrition Department, School of Medicine, University Rovira i Virgili, Reus, Tarragona, Spain; 5 Department of Preventive Medicine, School of Medicine, University of Valencia, Valencia, Spain; 6 Cardiovascular Risk and Nutrition Research Group, Institut Municipal d'Investigació Medica (IMIM)-Institut de Recerca del Hospital del Mar, Barcelona, Spain; 7 CIBER Epidemiología y Salud Pública (CIBERESP), Instituto de Salud Carlos III (ISCIII), Spanish Government, Madrid, Spain; 8 Department of Nutrition and Food Science, School of Pharmacy, University of Granada, Granada, Spain; 9 Institute of Nutrition and Food Technologies, University of Granada. Armilla, Granada, Spain; 10 Lipid Clinic, Endocrinology and Nutrition Service, Hospital Clinic, l'Institut d'Investigacions Biomèdiques August Pi i Sunyer (IDIBAPS), Barcelona, Spain; 11 Group of Nutrition and Lipid Metabolism, Instituto de la Grasa (CSIC), Seville, Spain; 12 Department of Preventive Medicine and Public Health, School of Medicine – Clinica Universitaria de Navarra, University of Navarra, Pamplona, Navarra, Spain; 13 Department of Preventive Medicine, School of Medicine, University of Malaga, Malaga, Spain; 14 Department of Family Medicine, Primary Care Division of Sevilla, San Pablo Health Center, Sevilla, Spain; 15 Internal Medicine Service, Hospital of Bellvitge, Barcelona, Spain; 16 Department of Cardiology, Hospital Txagorritxu, Vitoria, Alava, Spain; 17 University Institute for Health Sciences Investigation, University of Balearic Islands, Palma de Mallorca, Spain; 18 Department of Nutrition and Bromatology, School of Pharmacy, University of Barcelona, Barcelona, Spain; 19 Department of Cardiology, Hospital Son Espases, Palma de Mallorca, Spain; Geisel School of Medicine at Dartmouth College, United States of America

## Abstract

**Objective:**

Different types of carbohydrates have diverse glycemic response, thus glycemic index (GI) and glycemic load (GL) are used to assess this variation. The impact of dietary GI and GL in all-cause mortality is unknown. The objective of this study was to estimate the association between dietary GI and GL and risk of all-cause mortality in the PREDIMED study.

**Material and Methods:**

The PREDIMED study is a randomized nutritional intervention trial for primary cardiovascular prevention based on community-dwelling men and women at high risk of cardiovascular disease. Dietary information was collected at baseline and yearly using a validated 137-item food frequency questionnaire (FFQ). We assigned GI values of each item by a 5-step methodology, using the International Tables of GI and GL Values. Deaths were ascertained through contact with families and general practitioners, review of medical records and consultation of the National Death Index. Cox regression models were used to estimate multivariable-adjusted hazard ratios (HR) and their 95% CI for mortality, according to quartiles of energy-adjusted dietary GI/GL. To assess repeated measures of exposure, we updated GI and GL intakes from the yearly FFQs and used Cox models with time-dependent exposures.

**Results:**

We followed 3,583 non-diabetic subjects (4.7 years of follow-up, 123 deaths). As compared to participants in the lowest quartile of baseline dietary GI, those in the highest quartile showed an increased risk of all-cause mortality [HR = 2.15 (95% CI: 1.15–4.04); P for trend  = 0.012]. In the repeated-measures analyses using as exposure the yearly updated information on GI, we observed a similar association. Dietary GL was associated with all-cause mortality only when subjects were younger than 75 years.

**Conclusions:**

High dietary GI was positively associated with all-cause mortality in elderly population at high cardiovascular risk.

## Introduction

The epidemiological transition and increased prevalence of chronic-degenerative diseases in the elderly population is related to nutritional status. In Spain, 35% of the population aged 65 years or more is obese [1]. Obesity, especially visceral obesity, confers an increased risk of mortality not only from cardiovascular diseases (CVD) but also from other causes such as cancer or diabetes and its complications [2].

Currently, dietary recommendations from FAO and WHO to prevent chronic diseases are aimed to reduce or change the proportion of fats to a healthier profile, while increasing the carbohydrate content of the diet [2]–[5]. Although these recommendations establish a limit in the intake of free sugars and suggest the ideal amount of fiber consumption, they are not specific in relation to the quality of the dietary carbohydrates [2]–[5]. New evidence-based recommendations from the WHO are being prepared and probably further adjustments will be made regarding the proportion of fat and sugar intake. However, both the quality and the amount of carbohydrates, generate different responses of insulin secretion and postprandial glucose [6]. Thus, Jenkins et al. [7] introduced the concept of glycemic index (GI). GI is defined as the postprandial blood glucose response of a test food, compared to the response of the same amount of available carbohydrate in a reference food consumed by the same subject. Similarly, glycemic load (GL) evaluates the overall glycemic effect of the diet, multiplying the glycemic index of the diet by the content of total carbohydrates consumed by the individual [8]. Dietary GI and GL were developed to measure the average daily consumption of an individual in terms of quality and amount of available carbohydrates [9].

Current research has evaluated the relationship between dietary GI and GL in the prevention and study of obesity, CVD, cancer and many other health problems, such as macular degeneration [6], [9]–[12].

The literature to confirm the relationship between GI and GL for overall fatal events is scarce. In fact, usually, references are related to specific causes of fatal events such as cancer [13] and cardiovascular disease mortality [14], [15]. The association between dietary GI and GL and the risk of total mortality has been investigated in men with established CVD [16] and breast cancer survivors [17], but no significant associations were found between all-cause mortality when comparing the highest intakes of dietary GI and GL vs. the lowest. Furthermore, an association between higher intake of dietary GL and risk for all-cause mortality has been found in diabetic subjects with normal weight [18]. Similarly, Baer et al. [19] found a relationship in women aged 30–55 years: an increase of 41 units of GL, augment by 22% the risk for total mortality.

The association between GI/GL and all-cause mortality is still unclear; therefore, we aimed to evaluate the association of the quality of carbohydrates on all-cause mortality in an elderly population at high cardiovascular risk.

## Materials and Methods

### Study design

This study was conducted within the frame of the PREDIMED study (PREvención con DIeta MEDiterránea), a randomized, multicenter, parallel group, single-blinded dietary intervention trial conducted in Spain (Trial Registration: clinicaltrials.gov Identifier: ISRCTN35739639) [20]. The main objective of the trial was to analyze the effect of the Mediterranean Diet (MeDiet) on major cardiovascular disease prevention when comparing to a control diet [20]. Participants were randomly assigned to three groups: MeDiet supplemented with extra virgin olive oil (MeDiet + EVOO), MeDiet with nuts (MeDiet + nuts), and the control group. Participants received dietary training without calorie restriction or advice on physical activity [20]. After the completion of the trial, the PREDIMED study provided an opportunity for conducting the long-term follow-up of a large observational cohort of high cardiovascular risk subjects in a Mediterranean setting [21]. The design of the PREDIMED study has been reported in detail elsewhere [21]. The protocol was approved by the institutional review boards of each recruitment center and all participants provided a written informed consent prior to their inclusion in the study.

### Study population

The PREDIMED study included 7447 participants, aged between 55 and 80 years. Subjects without prior CVD were selected when they met at least one of the following criteria: presence of type 2 Diabetes Mellitus (T2DM) or the presence of three or more cardiovascular risk factors (current smoking, hypertension, high LDL cholesterol (≥160 mg/dl), low HDL cholesterol (≤40 mg/dL in men and ≤50 mg/dL in women), overweight or obesity (BMI≥25 kg/m^2^), or family history of premature CVD).

For this analysis, we selected participants without T2DM at baseline (n = 3833). Exclusion criteria were: subjects without follow-up (n = 125), with values of total energy intake outside of predefined limits (<800 or>4000 kcal/d in men and: <500 or>3500 kcal/d in women) (n = 86), use of anti-diabetic medication (n = 21) and incomplete data in any variable used in the analyses (n = 18). Overall, 3,583 subjects were analyzed in this study. The rationale for exclusion of persons with a baseline history of diabetes mellitus (including type 1 diabetes, T2DM or use of anti-diabetic medication) was potential effect modification and potential changes in the diet as a result of diagnosis and treatment of T2DM.

### Dietary assessment

Trained dietitians used a 137–item food frequency questionnaire (FFQ) to assess dietary habits by face-to-face interviews. This FFQ was repeatedly administered each year during follow-up. The FFQ has been validated in a sample of subjects with similar characteristics to the participants of the PREDIMED study [22]. Energy (kcal/day) and nutrient intake (g/day) were calculated as frequency multiplied by nutrient composition of specified portion size where frequencies were measured in nine categories for each food item. Nutrient data bank was updated using the latest available information included in food composition tables for Spain [23]. Alcohol intake was also ascertained through the use of this questionnaire. Carbohydrates, proteins, fat and dietary fiber intakes, GI and GL were adjusted for total energy intake using the residual method proposed by Willet [24].

### Estimation of dietary GI and GL

We assigned the GI of each food of the FFQ using Louie et al. protocol [25] with the International Tables of GI and GL values [26] and the Sydney University GI research service [27]. Values were extracted from published studies conducted in normal subjects, using glucose as reference food [26].

After the GI assignment, we estimated dietary GI for each individual summing the GI of each food multiplied by the amount of available carbohydrate consumed (g/day) and divided by the total available carbohydrate amount. For dietary GL, the sum of the GI multiplied by the amount of available carbohydrate consumed was divided by 100 [28]. Energy-adjusted dietary GI and GL intake was finally categorized into quartiles. To assess the repeated measurements of diet, we updated GI and GL values from the yearly FFQs.

### Other measurements

Socio-demographic information, medical history and lifestyle habits were collected via specific questionnaires carried out by trained personnel. Height and weight were measured wearing light clothes, barefoot, using a wall-mounted stadiometer and calibrated scales. BMI was estimated as weight (kg) divided by the height (m^2^) squared. We estimated energy expenditure using a validated Spanish version of the Minnesota leisure-time physical activity questionnaire [29], [30].

### Outcome ascertainment

All-cause mortality was determined by review of the End Point adjudication Committee. This panel was blinded to the intervention group. Information on all-cause mortality was updated on a yearly basis. Information on the occurrence of each fatality was initially obtained from the continuous contact with participants and their families that we had during the trial, contact with family physicians, the yearly comprehensive review of all medical records and by yearly consultation of the National Death Index. The analyses included cases confirmed between October 1st, 2003, and December 1st, 2010.

### Statistical analysis

Baseline characteristics of the population and dietary intakes were calculated according to quartiles of dietary GI and GL. Follow up time was calculated from the date of recruitment to the date of either death or end of follow-up (the date of the last visit or the last recorded clinical event of participants still alive). Different Cox regression models were used to estimate multivariable-adjusted hazard ratios (HR) and their 95% CI for all-cause mortality according to baseline quartiles of energy adjusted dietary GI and GL. A first model was adjusted for sex, age (years), recruitment center and intervention group (Med Diet + EVOO, Med Diet + Nuts and control diet). A second model was further adjusted for potential confounders including: smoking (never, past, current), education (low, middle, high, no data available), marital status (married, other), physical activity (continuous), BMI (continuous), self-reported history of cancer (yes, no), self-reported history of arterial hypertension (yes, no), self-reported history of dyslipidemia (yes, no), self-reported history of cardiovascular disease (yes, no), total energy intake (continuous), alcohol intake (continuous) and dietary fiber intake (continuous, energy adjusted). The third model was further adjusted for saturated fatty acids (continuous, energy adjusted) and monounsaturated fatty acids (continuous, energy adjusted). In all the analyses, recruitment center was entered into the model through the strata statement. Tests of linear trend across increasing quartiles of GI and GL were conducted by assigning the medians to each quartile and these variables were treated as continuous in the multivariate models. To assess a possible interaction between dietary GI/GL and sex or intervention group, we introduced product terms in the different multivariable models and considered p<0.05 in the likelihood ratio test as statistically significant.

To update GI and GL intake with all available longitudinal data, we used Cox regression models with time-dependent exposures. Three models were adjusted using the same covariates used for the models of baseline analysis. For dietary measures, we used the cumulative average of food intakes from baseline to the censoring events. BMI and physical activity were also yearly updated. Because changes in diet after development of diabetes may confound the association between exposure and mortality [31], we stopped updating dietary variables at the time interval during which individuals developed T2DM.

Finally, sensitivity analyses were conducted using quartiles of energy-adjusted GI and GL specific for different groups of the population to observe the effect of changing energy limits (percentiles 1 and 99, percentiles 5 and 95), including population with T2DM, excluding obese subjects (BMI≥30 kg/m^2^) and including only obese subjects, excluding participants with more than 6 years of follow-up and exclusion of subjects aged 75 years or more. Statistical analyses were performed using Stata 12.0 (StataCorp, College Station, TX, USA) and the significance level was set at p<0.05.

## Results

In this study, participants were followed for a median of 4.7 years. Of 3,583 subjects, 123 cases of all-cause mortality were registered. The mean (SD) dietary GI and GL of participants at recruitment was 57.6 (4.7) and 117.6 (24.1) respectively. **Table 1** shows the main baseline characteristics of the population according to energy adjusted dietary GI and GL quartiles. Subjects in the top quartile of dietary GI differed from the individuals in the lowest quartile in that they were younger and more often male, were more likely to be current smokers and more likely to be married. Participants in the highest quartile of dietary GI also had slightly lower BMI (−0.6 kg/m^2^), higher consumption of alcohol and carbohydrate but less intake of protein, fat and dietary fiber than the subjects in the bottom quartile of dietary GI. Characteristics according to dietary GL were similar to GI excepting that subjects in the highest quartile of GL were more often female, older, less likely to be current smokers, more likely to have only primary education, less likely to be married and physically active, had lower alcohol consumption, lower energy intake had higher intake of fiber when compared to the lowest quartile. The proportion of prevalent diseases such as cancer or arterial hypertension was similar across quartiles of dietary GI and GL. According to intervention group, mean dietary GI and GL were significantly higher when comparing the highest quartiles vs. the lowest.

**Table 1:** Characteristics of non diabetic subjects in the PREDIMED study assessed at baseline according to quartiles of energy adjusted dietary glycemic index and dietary glycemic load^a^^,^ b.

	Energy adjusted dietary glycemic index					Energy adjusted dietary glycemic load				
Variables	Q1	Q2	Q3	Q4	p-value	Q1	Q2	Q3	Q4	p-value
	n = 896	n = 896	n = 896	n = 895		n = 896	n = 896	n = 896	n = 895	
**Demographic characteristics**										
**Sex (% Female)**	75.8	67.1	57.5	48.9	<0.001	56.1	66.6	65.5	61.0	<0.001
**Age (years)**	66.8±5.9	67.0±6.2	66.9±6.1	66.0±6.1	0.007	65.8±6.0	67.1±5.8	66.7±6.1	67.0±6.4	<0.001
**Smoking (%)**					<0.001					<0.001
** Current**	12.7	11.4	16.5	22.0		19.4	14.4	12.8	16.0	
** Past**	18.2	20.1	22.8	27.2		24.9	21.2	21.7	20.5	
** Never**	69.1	68.5	60.7	50.8		55.7	64.4	65.5	63.6	
**Education (%)**					0.547					<0.001
** Elementary school**	73.9	73.8	73.6	73.5		67.5	73.9	74.0	79.3	
** Secondary school**	15.6	17.0	16.3	16.4		20.9	16.0	16.7	11.7	
** Graduate school**	8.4	7.4	7.7	9.1		9.8	8.5	6.7	7.5	
** No data available**	2.1	1.9	2.5	1.0		1.8	1.7	2.6	1.5	
**Marital status (% Married)**	72.2	78.0	75.2	78.1	0.010	82.4	76.7	72.5	72.0	<0.001
**Physical activity (METs-h/w)**	233.3±229.4	227.2±237.1	218.5±217.1	215.3±209.0	0.300	251.1±236.9	201.1±204.6	223.6±224.3	218.4±224.2	<0.001
**BMI (kg/m^2^)**	30.3±3.7	30.1±3.6	30.1±3.6	29.7±3.5	0.013	30.3±3.7	30.1±3.8	29.9±3.6	29.8±3.5	0.032
**Cancer (%)**	3.6	2.7	3.4	3.5	0.712	3.8	2.6	2.8	3.9	0.703
**Arterial hypertension (%)**	91.6	92.0	92.0	91.0	0.849	91.0	91.3	91.9	92.4	0.262
**Alcohol intake (g/d)**	6.0±11.2	7.5±12.7	10.3±16.3	12.1±16.9	<0.001	15.0±20.5	7.6±11.8	7.1±11.7	6.1±10.8	<0.001
**Dietary intake**										
** Total energy intake (kcal/d)**	2263±536	2251±525	2297±516	2284±531	0.257	2394±507	2177±485	2177±524	2346±555	<0.001
** Carbohydrate intake (g/d)** c	235.3±39.1	241.8±39.1	245.0±39.6	251.9±40.0	<0.001	199.1±27.2	232.5±16.9	253.5±17.9	288.9±27.8	<0.001
** Protein intake (g/d)** c	95.6±14.0	91.4±12.9	89.3±13.0	86.1±12.1	<0.001	93.9±15.3	90.7±12.6	90.3±12.7	87.6±12.2	<0.001
** Fat intake(g/d)** c	100.8±16.2	98.6±16.8	96.2±15.8	93.1±16.6	<0.001	111.6±14.5	102.4±10.6	93.6±10.7	81.1±12.7	<0.001
** Monounsaturated fatty acids (g/d)** c	49.3±10.7	48.9±11.4	48.2±10.6	47.4±10.6	0.005	56.3±10.1	51.5±8.4	46.2±8.4	39.7±8.7	<0.001
** Polyunsaturated fatty acids (g/d)** c	16.0±5.3	15.8±4.8	15.5±4.7	14.7±4.6	<0.001	17.8±5.4	16.2±4.3	15.1±4.4	12.9±4.2	<0.001
** Saturated fatty acids (g/d)** c	25.9±5.9	25.0±5.6	24.2±5.4	23.2±5.5	<0.001	28.0±5.9	25.8±4.7	23.8±4.3	20.7±5.0	<0.001
** Dietary fiber intake (g/d)** c	27.6±8.2	26.5±7.6	24.9±7.4	22.7±6.9	<0.001	23.1±6.9	24.6±6.4	26.3±7.6	27.8±9.1	<0.001
** GI (per day)** c	51.7±2.2	55.9±0.9	59.0±0.9	63.6±2.4	<0.001					
**GL (per day)** c						88.5±12.2	109.4±4.2	124.0±4.5	148.6±15.0	<0.001
	**Mean GI** c **(SD) according to intervention group**					**Mean GL** c **(SD) according to intervention group**				
** Mediterranean Diet + EVOO**	51.4±2.3	55.9±1.0	59.1±0.9	63.6±2.6	<0.001	88.5±11.4	109.3±4.2	124.2±4.5	148.9±16.2	<0.001
** Mediterranean Diet + Nuts**	51.8±2.2	55.9±0.9	59.0±1.0	63.5±2.1	<0.001	88.1±13.3	109.3±4.4	123.9±4.5	148.3±14.2	<0.001
** Control group**	51.8±2.0	56.0±0.9	59.0±1.0	63.8±2.6	<0.001	89.0±11.5	109.7±3.9	124.0±4.6	148.6±14.3	<0.001

a Data are presented as mean ± SD for quantitative variables and as percentages for qualitative variables, n = 3604.

b Abbreviations: n, number of subjects; Q, quartile; MET, metabolic equivalent; BMI, body mass index; GI, dietary glycemic index; GL, dietary glycemic load.

c Values are adjusted for energy using the residuals method.

We found a significant association between the highest quartile of dietary GI and all-cause mortality (**Table 2**). In the first model, subjects in the top quartile of baseline dietary GI had a 2.2 fold risk of all-cause mortality vs. subjects in the lowest quartile [HR = 2.22 (95% CI: 1.26–3.94); p for trend  = 0.002]. After multivariate adjustment, this association was attenuated but remained significant [HR = 2.15 (95% CI: 1.15–4.04); P for trend  = 0.012]. Our results revealed a higher risk for all cause mortality and baseline dietary GL in the multivariate analysis although it did not reach statistical significance [HR = 1.95 (95% CI: 0.97–3.90); p for trend  = 0.072]. No significant interactions with sex or intervention group were observed. In the repeated measurement analyses using as exposure the yearly updated information on GI, we found a significant association with all-cause mortality [HR = 2.25 (95% CI: 1.16–4.36) for the highest versus the lowest quartile; p for trend  = 0.014]. Regarding updated dietary GL, we observed that subjects in the top quartile had around 75% higher risk for mortality when compared to those in the bottom quartile, however, this association did not achieve statistical significance.

**Table 2:** Hazard Ratios (95% CI) for total mortality by quartiles of energy adjusted dietary glycemic index and dietary glycemic load assessed at baseline in non-diabetic subjects^a^.

	Baseline energy adjusted dietary glycemic index					Baseline energy adjusted dietary glycemic load				
	Q1	Q2	Q3	Q4	p-trend	Q1	Q2	Q3	Q4	p-trend
**Median**	52.1	55.9	59.1	63.1		91.9	109.6	123.9	144.4	
**Cases**	17	23	32	51		33	28	22	40	
**Person-years**	3797	3830	3925	4003		4010	3948	3862	3735	
**HR** b **(95% CI)**	1 (Ref.)	1.17 (0.62–2.19)	1.47 (0.81–2.67)	2.22 (1.26–3.94)	0.002	1 (Ref.)	0.97 (0.59–1.58)	0.80 (0.47–1.36)	1.43 (0.88–2.32)	0.210
**HR** c **(95% CI)**	1 (Ref.)	1.30 (0.68–2.49)	1.40 (0.75–2.61)	2.00 (1.08–3.70)	0.019	1 (Ref.)	1.04 (0.61–1.77)	0.90 (0.49–1.65)	1.75 (1.00–3.06)	0.070
**HR** d **(95% CI)**	1 (Ref.)	1.36 (0.72–2.58)	1.46 (0.79–2.70)	2.15 (1.15–4.04)	0.012	1 (Ref.)	1.09 (0.63–1.88)	0.96 (0.51–1.82)	1.95 (0.97–3.90)	0.072

	Updated energy adjusted dietary glycemic index					Updated energy adjusted dietary glycemic load				
**HR** b **(95% CI)**	1 (Ref.)	1.32 (0.70–2.48)	1.63 (0.88–3.02)	2.69 (1.50–4.84)	0.001	1 (Ref.)	1.49 (0.91–2.44)	0.91 (0.53–1.59)	1.41 (0.86–2.30)	0.381
**HR** e **(95% CI)**	1 (Ref.)	1.26 (0.66–2.41)	1.52 (0.80–2.91)	2.18 (1.13–4.20)	0.015	1 (Ref.)	1.67 (1.01–2.77)	1.09 (0.61–1.95)	1.73 (0.98–3.05)	0.212
**HR** f **(95% CI)**	1 (Ref.)	1.27 (0.67–2.43)	1.55 (0.81–2.96)	2.25 (1.16–4.36)	0.014	1 (Ref.)	1.69 (0.98–2.91)	1.10 (0.59–2.08)	1.76 (0.88–3.54)	0.326

a Abbreviations: Q, quartile; HR, hazard ratio; CI, confidence interval.

b Adjusted for sex, age (years), recruitment center, intervention group (Med Diet with EVOO, Med Diet with Nuts, Low fat diet).

c Adjusted for sex, age (years), recruitment center, intervention group (Med Diet + EVOO, Med Diet + Nuts, Low fat diet), smoking (never, past, current), education (low, middle, high, no data available), marital status (married, other), physical activity (continuous), BMI (continuous), self-reported history of cancer (yes, no), self-reported history of arterial hypertension (yes, no), total energy intake (continuous), alcohol intake (continuous) and dietary fiber intake (continuous, energy-adjusted).

d Multivariate model with additional adjustment for intake of saturated fatty acids (continuous, energy-adjusted) and monounsaturated fatty acids (continuous, energy-adjusted).

e Multivariate model with yearly updated measures of physical activity (continuous), BMI (continuous), total energy intake (continuous), alcohol intake (continuous), dietary fiber intake (continuous, energy-adjusted).

f Multivariate model with yearly updated measures with additional adjustment for saturated fatty acids intake (continuous, energy-adjusted) and monounsaturated fatty acids intake (continuous, energy-adjusted).

Results from sensitivity analyses comparing the risk of mortality between the upper quartiles (quartile 3 and quartile 4) of GI and GL with reference to the lowest quartile are shown in **Table 3**. We also found positive associations between the upper quartile of dietary GI with all-cause mortality when considering energy limits as percentiles 1 and 99 or percentiles 5 and 95, and in subjects younger than 75 years. Finally, obese subjects in the top quartiles of dietary GI presented a 3-fold increased risk for all-cause mortality with a statistically significant positive trend (p for trend  = 0.044). Dietary GL was associated with all-cause mortality when we excluded participants with total energy intake lower than percentile 1 or higher than percentile 99 [HR = 2.10 (95% CI: 1.03–4.27) for the highest versus the lowest quartile; p for trend  = 0.042] and when we restricted the analysis to subjects younger than 75 years [HR = 3.16 (95% CI: 1.32–7.54); p for trend  = 0.019].

**Table 3:** Sensitivity analysis. Hazard Ratios (95% CI) for total mortality according to baseline quartiles of energy adjusted dietary glycemic index and dietary glycemic load^a^.

		Energy adjusted dietary glycemic index^b^			Energy adjusted dietary glycemic load^b^		
	n	Q3	Q4	p-trend	Q3	Q4	p-trend
**Energy limits percentiles 1 and 99**							
HR^b^ (95% CI)	3606	1.47 (0.80–2.71)	2.08 (1.12–3.87)	0.015	1.14 (0.60–2.16)	2.10 (1.03–4.27)	0.042
**Energy limits percentiles 5 and 95**							
HR^b^ (95% CI)	3333	1.46 (0.76–2.80)	2.39 (1.23–4.63)	0.010	0.86 (0.43–1.71)	1.96 (0.94–4.06)	0.109
**Sample with diabetic population**							
HR^b^ (95% CI)	7013	0.94 (0.68–1.30)	1.21 (0.86–1.71)	0.272	1.02 (0.70–1.49)	1.16 (0.76–1.78)	0.657
**Excluding obese subjects (BMI≥30 kg/m^2^)**							
HR^b^ (95% CI)	1888	0.76 (0.35–1.65)	1.79 (0.83–3.86)	0.221	0.79 (0.33–1.90)	1.94 (0.76–4.97)	0.214
**Including only obese subjects (BMI≥30 kg/m^2^)**							
HR^b^ (95% CI)	1695	3.75 (1.11–12.64)	3.24 (0.96–10.86)	0.044	1.25 (0.46–3.42)	2.10 (0.69–6.38)	0.183
**Excluding subjects>6 y of follow-up**							
HR^b^ (95% CI)	2935	1.13 (0.61–2.08)	1.66 (0.89–3.09)	0.052	0.81 (0.42–1.56)	1.75 (0.88–3.45)	0.160
**Excluding subjects ≥75 y**							
HR^b^ (95% CI)	3158	1.46 (0.67–3.18)	2.35 (1.04–5.33)	0.026	1.45 (0.66–3.19)	3.16 (1.32–7.54)	0.019

a Abbreviations: Q, quartile; HR, hazard ratio; CI, confidence interval; BMI, Body Mass Index.

b Model adjusted for sex, age (years), recruitment center, intervention group (Med Diet + EVOO, Med Diet + Nuts, Low fat diet), smoking (never, past, current), education (low, middle, high, no data available), marital status (married, other), physical activity (continuous), BMI (continuous), self-reported history of cancer (yes, no), self-reported history of arterial hypertension (yes, no), total energy intake (continuous), alcohol (continuous), dietary fiber intake (continuous, energy-adjusted), saturated fatty acids intake (continuous, energy-adjusted) and monounsaturated fatty acids intake (continuous, energy-adjusted).

b Dietary GI and GL quartiles were estimated for each specific population group.

## Discussion

We found that higher intake of baseline dietary GI was associated with an increased risk of all-cause mortality in 3,583 non-diabetic elderly subjects. This association remained significant in the repeated measurement analysis. With respect of dietary GL, we observed a higher risk of all-cause mortality only in subjects at high risk of CVD younger than 75 years.

Our results differ from a cohort of Swedish men, aged from 45 to 79 years with prior cardiovascular disease, where no association was found between dietary GI nor GL and all-cause mortality [16]. We did not find a relationship for all-cause mortality and dietary GL for the original sample evaluated; however, the association was statistically significant for subjects younger than 75 years. This effect is similar to the Nurses' Health Study, where GL was identified as a risk factor for all-cause mortality among 50,112 women aged 30–55 years [19]. It is of interest to note that in the Nurses' Health study a significant association was found with “other causes” but not with CHD and cancer mortality. Therefore, the differences could be explained because our study was conducted in aged population at high cardiovascular risk and our endpoint incorporated all causes of death, including CHD and cancer.

Our results also agree with a previous study where Oba et al. reported an association of similar magnitude between dietary GI and mortality from stroke risk in women. No associations were observed between dietary GL and the risk of total stroke or death from ischemic stroke [15]. A positive trend was found for GL and death from hemorrhagic stroke in women. In our study, a significant trend was found in the sensitivity analysis only in subjects younger than 75 years. When individuals over 75 years were analyzed, no associations were found between dietary GI nor GL with all cause mortality (data not shown). This fact reflects that the oldest subjects in our sample could be attenuating our results due to age-related influences, such as deterioration in glucose metabolism. Pancreatic, insulin receptor, and post-receptor changes associated with aging are critical components of the endocrinology of aging. Apart from decreased (relative) insulin secretion by the β cells, peripheral insulin resistance related to poor diet, physical inactivity, increased abdominal fat mass, and decreased lean body mass contribute to the deterioration of glucose metabolism [32]. Furthermore, this age-effect is similar to previous findings, where the association between obesity and mortality was attenuated with advancing age [33]. However the age-related weaker associations may reflect confounding from cohort variation in mortality risk, healthy participant effects (i.e., biases introduced by survey selection of healthy respondents) or duration of exposition to the risk factor [34]. Therefore, we consider that it can be not concluded whether dietary GL is associated with higher risk of mortality in older subjects and further studies with a larger sample size and follow-up are needed to confirm the association between dietary GL and all-cause mortality. Our results alternatively, may indicate that, in this population at high cardiovascular risk, the quality of carbohydrates (represented by dietary GI) could be more important than its quantity (partially represented by dietary GL) in predicting all-cause mortality [15].

We identified an association between the highest quartiles of dietary GI and risk of all-cause mortality in obese subjects. Two recent meta-analyses have shown that the effect of dietary GL on the risk of coronary heart disease appeared more evident in subjects with higher BMI, although the authors recommended treating this information with caution because of the limited evidence and diversity among the BMI cut-off points used across studies [35], [36]. Nevertheless, the individual response to a given carbohydrate load is influenced by the degree of insulin resistance, which is, firstly determined by the degree of adiposity, and also by physical activity, genetics, and other aspects of diet. Thus, it might be expected that the adverse metabolic effects of high-GI foods would be exacerbated in sedentary, overweight, or genetically susceptible persons [37].

Furthermore, higher values of dietary GI or GL could increase the risk of all-cause mortality by raising the risk of chronic diseases. Recent meta-analyses have shown a relationship between dietary GI, GL and increased risk of CVD in women but not in men [35], [38], [39], greater risk of T2DM [6], [40], [41], and risk for certain types of cancer such as colorectal or endometrial ones [11], [42]. Higher dietary GI has also been associated with an increased risk of breast cancer [6], [11], [43] although these results are contradictory [44]–[46].

After a high-GI meal, blood glucose concentration increases at least twice that after a low-Gl meal with the same nutrients and energy. This hyperglycemia stimulates insulin release and inhibits glucagon liberation. High insulin-to-glucagon ratio affects normal anabolic responses such as uptake of nutrients by insulin-responsive tissues, glycogenesis, lipogenesis, and inhibits gluconeogenesis and lipolysis. From 2 to 4 hours after a high-GI meal, the absorption from the gastrointestinal tract declines, but the effects of hiperinsulinemia and low glucagon remain. Blood glucose drops to lower hypoglycemic range and release of free fatty acid is more suppressed than compared with a low-GI meal. In the late postprandial period, the low circulating concentration of metabolic fuel activates the hormone response that restores euglicemia and elevates free fatty acids to higher levels than observed after low-GI meals [47].

Eventually these postprandial responses may contribute to insulin resistance and obesity [48]. In a meta-analysis, Livesey et al. found that reduction in GL was associated with a decrease in body weight and vice versa [49]. When comparing high GI foods, such as white bread, Bautista-Castaño et al. found a dose-response relationship between the increase in white bread consumption and weight or waist circumference gain [50]. However, these results are inconsistent. A systematic review of 14 randomized controlled trials did not find differences in the effect of low GI/GL vs. high GI/GL diets on anthropometric data, but decreases in C-reactive protein and fasting insulin were significant in the low GI/GL groups [51].

Additionally, higher dietary GI has been associated with small increases in C-reactive protein [52] which has been related to all-cause mortality [53]. Another European study conducted in overweight subjects, showed that following a low GI diet after a weight loss intervention, had a greater decrease of high sensitivity C-reactive protein blood levels than the high GI groups [54]. Moreover, in the PREDIMED study, top quartiles of dietary GI were associated with higher plasma levels of TNF and IL-6 than those in the lowest quartiles [55].

A study conducted in healthy elderly Europeans found that a posteriori plant-based dietary pattern was associated with lower all-cause mortality. This plant-based pattern with high intakes of vegetables, vegetable oils, fruit, legumes and pasta/rice/other grains and low intakes of potatoes, margarine and non-alcoholic beverages, was correlated with the MeDiet pattern [56]. A previous study has shown that greater adherence to the MeDiet is inversely associated with dietary GI and GL [57]. A high degree of adherence to the MeDiet has been associated with a reduction in total mortality [58]–[62], obesity [63]–[65], T2DM [66], [67], major cardiovascular events [21], [68] and their risk factors [23]. Thus, Estruch et al. [23] assessed the effects of a 3-month intervention with MeDiet on changes in cardiovascular risk factors within the PREDIMED trial. MeDiet supplemented with EVOO, reduced C-reactive protein levels. Moreover, other inflammation biomarkers such as interleukin-6, intercellular adhesion molecule-1 (ICAM-1) and vascular cell adhesion molecule-1 (VCAM-1) decreased in subjects following a MeDiet supplemented either with EVOO or nuts [23]. Even more, after one year of intervention with MeDiet, the prevalence of metabolic syndrome was reduced by 13.7% when compared with control group [69]. However the number of randomized controlled-feeding trials that compare low vs. high GI/GL diets and that include measures of glucose homeostasis, blood lipids or inflammation is limited [70].

The limitations of this study are mainly methodological. Firstly, due to the scarcity of GI values for Spanish foods, we used as reference GI data from other countries. This fact could be a source of bias, because GI values may differ wide ranges, depending on variety, processing and cooking [71]. Secondly, the FFQ was not designed to evaluate dietary GI and dietary GL. Thirdly, we conducted the study in elderly subjects at high cardiovascular risk. Therefore, our results cannot be generalized to other populations. Finally, our study was conducted in a cohort that went through a nutritional intervention, which may have had an effect on dietary GI and GL. However, in order to address this issue, we adjusted all analyses by intervention group to minimize the effect. Moreover, because updated dietary information during the follow-up was used in our analyses, we accounted for changes in dietary habits over time. To our knowledge, this is the first large cohort study assessing GI and GL with yearly repeated measurements of diet. Repeated measurements of diet capture changes in dietary exposure, but also contribute to overcome, at least partially, the potential problems of measurement errors in nutritional epidemiology. Our study also has other strengths, such as the large sample size that allowed us to adjust for all possible potential confounders in the multivariate analyses. Other strengths are the use of a comprehensive and validated FFQ and the assignment of GI values trough an established protocol.

## Conclusions

In summary, this study provides evidence that high GI diets are related to increased risk of all-cause mortality in non-diabetic elderly subjects at high cardiovascular risk. In the sensitivity analyses this association was statistically significant when subjects were younger than 75 years or obese. Nevertheless, more evidence is necessary to evaluate these associations in different population groups and further studies that elucidate the mechanisms supporting the inclusion of GI and GL in dietary recommendations.
